# Portal Cholangiopathy: An Uncommon Cause of Right Upper Quadrant Pain

**DOI:** 10.7759/cureus.10281

**Published:** 2020-09-06

**Authors:** Vikram B Itare, Donya Imanirad, Abdulaziz Almaghraby

**Affiliations:** 1 Internal Medicine, Smolensk State Medical University, Smolensk, RUS; 2 Internal Medicine, University of South Florida, Tampa, USA; 3 Internal Medicine Department, Umm Al-Qura University, Makkah, SAU

**Keywords:** portal cavernoma cholangiopathy, ruq pain

## Abstract

Portal cholangiopathy is one of the complications of the chronic portal vein thrombosis (PVT). Chronic PVT can occur in a patient with acute PVT that usually does not resolve regardless of the treatment. There is a development of collateral blood vessels that bring blood from the portal system towards the liver around the obstruction area, known as the cavernous transformation of the portal vein or portal cavernoma, in a patient with chronic PVT. The appearance and location of collateral channels depends on the extent and location of thrombus in the portomesenteric venous system. If the portomesenteric venous system is occluded near the formation of the portal vein, blood tends to flow through collateral channels that form varices in and around the common bile duct. Portal cholangiopathy (also referred to as portal biliopathy) is common in patients with long-standing chronic PVT. It is due to compression of the large bile ducts by the venous collaterals that form in patients with chronic PVT. Most of the patients with long-standing PVT have portal cholangiopathy. Typically, symptoms of portal cholangiopathy include jaundice, biliary colic, and pruritus. Portal cholangiopathy is a rare complication of chronic portal hypertension, and it is an important differential diagnosis of biliary colic secondary to cholelithiasis. The patient can also present with the sharp right upper quadrant pain, which is atypical by nature for biliary colic.

## Introduction

Hunt noted the earliest evidence of portal cholangiopathy in 1965 [1]. Meredith et al. and others reported a few cases of common bile duct obstruction caused by the extensive collateral venous circulation of the portal vein [2,3].

Portal cholangiopathy is an obstruction of the biliary tract in association with engorgement of collateral veins in the hepatic hilum [4]. When portal vein thrombosis (PVT) occurs, multiple venous collaterals develop to bypass the obstruction, which can cause extrinsic compression of the bile ducts. Portal cavernoma cholangiopathy consists of extrahepatic portal vein obstruction (EHPVO) and subsequent cavernous transformation of the portal vein. Most cases of EHPVO manifest as an extrinsic indentation on the bile duct and mild bile duct narrowing, but the majority are asymptomatic. However, progressive portal cavernoma cholangiopathy may lead to severe complications, such as secondary biliary cirrhosis.

Chronic PVT can lead to cavernous or variceal changes in the portal vein forming the collateral blood flow around the gallbladder, which usually leads to non-specific or ischemic changes in the gallbladder wall.

In general, the patients are asymptomatic, but jaundice, cholangitis, and biliary lithiasis may be present in 5% to 38% of patients [4-9]. Asymptomatic cases are detected by having biliary abnormalities either on endoscopic retrograde cholangiography (ERC) or magnetic resonance cholangiography (MRC) in the absence of any biliary symptoms. We describe a case of a 63-year-old man who presents with right upper quadrant (RUQ) pain for two days, secondary to portal cholangiopathy caused by chronic portal thrombosis and cavernous formation of the portal vein.

## Case presentation

A 63-year-old male patient with a past medical history of diabetes milieus, hypothyroidism, laryngeal cancer, hyperlipidemia, carotid artery stenosis, and a previous episode of pancreatitis presented to the emergency room (ER) with the RUQ pain. This pain bothered him for two days and woke him up from sleep. He characterized the pain as sharp, constant, waxing, and waning in intensity, worse with movement, and alleviated by staying still. He denied taking any medicine at home for pain control. The patient did not have nausea, vomiting, fever, or chills. He also denied any history of chronic non-steroidal anti-inflammatory drug (NSAID) or alcohol use. On physical examination, the patient had severe RUQ pain on superficial palpation but a negative Murphy sign.

**Figure 1:**
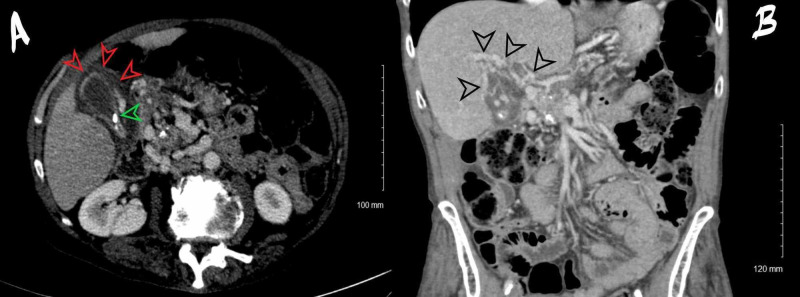
CT scan. (A) Axial CT scan through the gallbladder; gallbladder: two gallstones are present (green arrow). The gallbladder is not overly distended. There is pericholecystic fluid present (red arrows); pancreas: the pancreas is atrophic with parenchymal calcifications and prominence of the pancreatic duct. (B) Coronal reconstruction CT scan through porta hepatis shows normal caliber bile ducts. There is a cavernous transformation of the portal vein (black arrow). Numerous varices are identified, including perigastric and pericholecystic.

CT scan showed non-specific gallbladder wall edema, possibly related to the cavernous transformation of the portal vein around the pericholecystic area (Figure 1).

**Figure 2:**
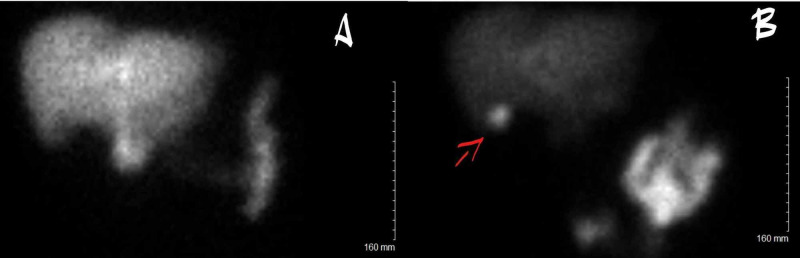
Hepatobiliary iminodiacetic acid (HIDA) scan The initial radioangiogram shows an excellent flow to the liver (not pictured). (A) Dynamic images show a uniform distribution of the tracer in the liver — excretion in the gut present by 15 minutes. The gallbladder is not visualized 60 minutes into the study. (B) At this time, additional 1.1 mCi of technetium 99m choletec is administered. Approximately 2 mg of morphine sulfate is given intravenously, and imaging is continued. The gallbladder is visualized following morphine administration (red arrow). Impression: No cystic or common duct obstruction. The gallbladder is visualized (red arrow) after 60 minutes and after morphine administration as above, findings suggestive of chronic gallbladder disease.

Hepatobiliary iminodiacetic acid (HIDA) scan showed no cystic or common duct obstruction. The gallbladder was visualized after 60 minutes and after morphine administration as above, findings suggestive of chronic gallbladder disease (Figure 2).

Laboratory investigations are summarized in Table 1 that revealed the initial derangement in the hepatic profile, which showed subsequent improvement. 

**Table 1:** Lab results.

Days	1	2	3	4	5
Aspartate aminotransferase	31	166 (H)	60 (H)	44	30
Alanine aminotransferase	43	251 (H)	153 (H)	104 (H)	76 (H)
Alkaline phosphatase	77	294 (H)	237 (H)	221 (H)	201 (H)

Subsequently, according to the diagnostic results, it was recommended against any operative procedures like cholecystectomy because of the high risk of hemorrhage due to collateral portal cavernoma formation, which is highly vascularized in the pericholecystic region, secondary to chronic PVT.

After a discussion of the risk of bleeding, it recommended starting anticoagulation with direct oral anticoagulation, apixaban, indefinitely as long as there are no severe bleeding issues to prevent recurrent thrombosis, thrombus extension, and development of portal hypertension.

Lastly, the patient was started on propranolol for the prevention of gastric variceal bleeding due to portal venous hypertension, which developed secondary to the chronic portal thrombosis. Propranolol will reduce the risk of variceal bleeding, which is the most common presenting feature of chronic portal hypertension.

Finally, the diagnosis of portal cholangiopathy was established based on CT scan findings showing gallbladder wall edema, possibly related to the cavernous transformation of portal vein around the pericholecystic area. Usually, the patients presenting with jaundice, recurrent biliary symptoms secondary to portal cholangiopathy, may benefit from endoscopic removal of bile duct stone followed by biliary stent insertion or creation of TIPS (transjugular intrahepatic portosystemic shunt). As this patient presented with the first episode of RUQ pain lasting for two days, with the concern of bleeding, all the surgical procedures were withheld. Our treatment approach followed using apixaban and propranolol.

## Discussion

Portal cholangiopathy is secondary to the cavernous formation of the portal vein in the pericholecystic region, causing the changes in the gallbladder wall. Cavernous or variceal changes in the portal vein are usually due to an increase in pressure and formation of collateral in the portal vein due to an increase in pressure and decrease in portal vein flow towards the liver secondary to the chronic PVT.

Biliary changes are present in about 77%-100% of patients with portal cholangiopathy [6,7,10]; however, only 5%-38% of patients developed biliary symptoms [7,10]. Symptoms and clinical manifestations of portal cholangiopathy can be related to chronic cholestasis or biliary stone formations, and they include jaundice, cholangitis, cholecystitis, abdominal pain, and cholelithiasis [11].

Risk factors for symptom occurrence in portal cholangiopathy are older age, longer duration of disease, common bile duct, gallbladder stones, and abnormal liver function tests (LFTs) [12].

In biliary colic, pain is neither exacerbated by movement nor relieved by squatting bowel movements or passage of flatus [13]. The pain typically lasts at least 30 minutes, plateauing within an hour. The pain then starts to subside, with an entire attack usually lasting less than six hours [14].

Most patients with chronic PVT are asymptomatic, and the diagnosis is identified incidentally by imaging done for other reasons. Patients with chronic PVT frequently have esophageal or gastric varices, and the most common clinical presentation is gastrointestinal bleeding [15]. In a retrospective series that included 40 patients with chronic newly diagnosed PVT, esophageal varices were present in 35 patients (88%), gastric varices in 20 patients (50%), portal hypertensive gastropathy in 19 patients (48%), and gastrointestinal bleeding in 19 patients (48%).

In summary, portal cholangiopathy is a rare complication of chronic portal hypertension, and it is a crucial differential diagnosis of biliary colic. Nevertheless, in this case, the patient presented with atypical features of biliary colic, including RUQ pain, was not post-prandial, which was aggravated due to movement. The duration of the pain was more than 48 hours.

## Conclusions

Jaundice and RUQ pain in this patient prompted the work-up for the cause. Portal cavernoma is an uncommon cause for these findings and can be an unrecognized diagnosis; hence, it should be included among the differential diagnoses for extrahepatic biliary obstruction.

Differential diagnosis of any patient with jaundice and RUQ pain should consider hepatic hilum neoplasia, such as cholangiocarcinoma or metastatic neoplasms, as well as other causes of intrahepatic bile ducts involvement, such as sclerosing cholangitis.

The gold standard for diagnosis of portal cavernoma is endoscopic retrograde cholangiopancreatography (ERCP). Doppler ultrasound studies can show gallbladder varices in patients. Patients with suspected portal biliopathy should be evaluated with MRC, and the results should be compared with the results of ERCP studies.

Morphological alterations in cholangiography can range from biliary tract dilatation associated with areas of stenosis and angulations to failure in duct filling due to compression by dilated parietal veins projecting towards the lumen.

Patients with symptoms and recurrent cholangitis can be treated using bile duct dilation. Before bile duct dilatation, portal decompression is recommended.
