# Correction: Portal Cholangiopathy: An Uncommon Cause of Right Upper Quadrant Pain

**DOI:** 10.7759/cureus.c369

**Published:** 2025-10-30

**Authors:** Vikram B Itare, Donya Imanirad, Abdulaziz Almaghraby

**Affiliations:** 1 Internal Medicine, Smolensk State Medical University, Smolensk, RUS; 2 Internal Medicine, University of South Florida, Tampa, USA; 3 Internal Medicine Department, University of South Florida, Umm Al-Qura University, SAU

The affiliation for Abdulaziz Almaghraby has been corrected from ‘Internal Medicine, University of South Florida, Tampa, USA’ to ‘Internal Medicine Department, Umm Al-Qura University, Makkah, SAU’.

